# Methiothepin Increases Chemotherapy Efficacy against Resistant Melanoma Cells

**DOI:** 10.3390/molecules26071867

**Published:** 2021-03-26

**Authors:** Nelly Durand, Méliné Simsir, Laurie Signetti, Fabien Labbal, Robert Ballotti, Isabelle Mus-Veteau

**Affiliations:** 1 Université Côte d’Azur, CNRS, IPMC, France, Institut de Pharmacologie Moléculaire et Cellulaire, 660 route des Lucioles, 06560 Valbonne, France; durand@ipmc.cnrs.fr (N.D.); simsir@ipmc.cnrs.fr (M.S.); lauriesignetti@gmail.com (L.S.); labbal@ipmc.cnrs.fr (F.L.); 2 Université Côte d’Azur, INSERM, Equipe Labellisée ARC, 2019, C3M, Bâtiment Universitaire Archimed 151 Route Saint Antoine de Ginestière BP 2 3194, CEDEX 3, 06204 Nice, France; Robert.Ballotti@unice.fr

**Keywords:** Ptch1, melanoma, vemurafenib, trametinib, chemotherapy resistance, Ptch1 drug efflux inhibitor, methiothepin

## Abstract

We previously reported that methiothepin, a small molecule known as a nonselective serotonin 5-HT receptor antagonist, inhibited the doxorubicin efflux activity of the Hedgehog receptor Ptch1 and enhanced the cytotoxic, pro-apoptotic, anti-proliferative, and anti-clonogenic effects of doxorubicin on adrenocortical carcinoma cells. Here, we show that methiothepin also inhibits doxorubicin efflux and increases doxorubicin cytotoxicity in melanoma cells which endogenously overexpress Ptch1. Melanoma patients having the BRAF^V600E^ mutation are treated with vemurafenib, an inhibitor of BRAF^V600E^, often in combination with trametinib, an inhibitor of MEK. Almost all patients ultimately acquire resistance to the treatment leading to disease progression. Here, we report that methiothepin overcomes the resistance of BRAF^V600E^ melanoma cells by enhancing the cytotoxicity of vemurafenib and trametinib on these cells leading to melanoma cells death. We observe that the addition of methiothepin to vemurafenib prevents migration of resistant melanoma cells more efficiently than vemurafenib alone. Our results provide an additional proof that Ptch1 drug efflux inhibition increases the effectiveness of anti-cancer treatments and overcomes resistance of melanoma cells expressing Ptch1.

## 1. Introduction

Cutaneous melanoma is a complex disorder characterized by elevated heterogeneity. It is one of the most aggressive types of cancer and one of the leading causes of skin cancer-related mortality, due to its metastatic power. Its therapeutic management is a real challenge, as it is amongst the solid malignancies most refractory to conventional cancer therapies [1]. Up to 90% of melanomas exhibit aberrant MAPK pathway activation that induces cell cycle deregulation and apoptosis inhibition, and marked improvements in cutaneous melanoma treatment have been achieved by targeting the MAPK signaling pathway. Improved overall survival outcomes were observed with targeted therapies in patients with BRAF^V600^ mutant unresectable stage III or stage IV melanoma. Nearly half of patients with metastatic melanomas harbor a valine to glutamine substitution in codon 600 of the serine/threonine kinase BRAF [2]. Vemurafenib, dabrafenib, and encorafenib are BRAF inhibitors (BRAFi) approved by the US Food and Drug Administration (FDA) to treat patients with BRAF^V600E^-mutated metastatic melanomas [3]. BRAFi have relatively high response rates; however, patients almost invariably develop disease progression after about five months. The addition of a MEK inhibitor such as trametinib to BRAFi extends the median duration of response from five months to nine months [4,5]. However, many patients develop resistance to BRAF (+/− MEK) inhibitors [5,6].

Amongst the mechanisms used by cancer cells to become resistant to treatment, multidrug resistance (MDR) has been intensively studied [7]. The most prominent mechanism underlying MDR is the overexpression of multidrug transporters, of which the best known in cancers were ATP-binding cassette (ABC) transporters [8]. However, to date, the FDA has not approved the use of any ABC transporter inhibitor, due to toxicity issues [9]. Therefore, counteracting chemotherapy resistance by alleviating MDR is still an unmet medical need.

We recently discovered that the Hedgehog receptor Ptch1, which is overexpressed in many cancers, has an efficient drug efflux function in cancer cells. We have demonstrated that Ptch1 pumps chemotherapeutic agents such as doxorubicin (dxr) which isused to treat many cancers, out of cancer cells that were derived from melanoma and adrenocortical carcinoma (ACC), thereby conferring resistance to chemotherapy [10,11,12]. Our analysis of normalized gene expression data and matching clinical information for cutaneous melanoma tumors downloaded from The Cancer Genome Atlas (TCGA) revealed that the Ptch1 protein was strongly expressed in metastatic samples from melanoma patients whose melanoma did or did not present the BRAF^V600^ mutation [12]. In this cohort, the Kaplan–Meier analysis for a subset of patients with metastatic disease who did not receive immunotherapy indicated that a high level of Ptch1 in patient samples significantly correlated with a lower overall survival time.

We developed screening tests to identify molecules able to inhibit the drug efflux activity of Ptch1, which led to the discovery of three inhibitors [13]. The first, panicein A hydroquinone (PAH), a compound purified from a marine sponge, increases the cytotoxicity of dxr and of the BRAF inhibitor vemurafenib against melanoma cells in vitro and in vivo [12,14]. The second inhibitor, methiothepin, a nonselective 5-HT receptor antagonist, increases the efficacy of dxr against adrenocortical carcinoma cells in vitro and in vivo [11]. The third inhibitor, astemizole, an anti-histaminergic drug, also increases the efficacy of dxr against adrenocortical carcinoma cells in vitro [15].

In the present study, we analyze the effect of methiothepin on melanoma cells. We show that methiothepin enhances the efficacy of doxorubicin against melanoma cells by binding Ptch1 and inhibiting Ptch1 efflux activity. We also report results demonstrating that methiothepin increases the effectiveness of the BRAF inhibitor vemurafenib and of the MEK inhibitor trametinib against resistant BRAF^V600E^ melanoma cells.

## 2. Results

### 2.1. Ptch1 Is Expressed in Melanoma Cells

**Figure 1:**
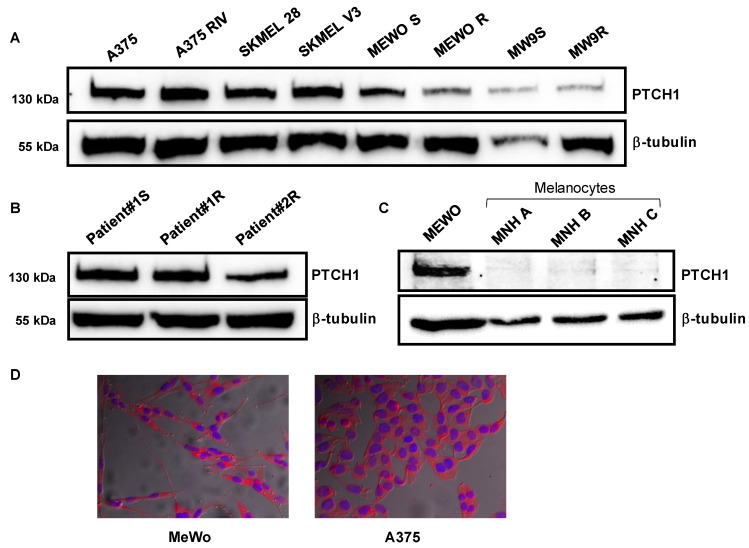
Ptch1 is expressed in various melanoma cell lines and in melanoma cells from patients. Western blots were performed on total extracts from several melanoma cell lines (**A**), melanoma cells from three patients sensitive or resistant to vemurafenib (**B**), and from melanocytes from three different primary cultures (**C**) using antibodies directed against Ptch1 and β-tubulin. (**D**) Immunofluorescence labeling of Ptch1 on MeWo and A375 cells with antibodies directed against Ptch1 (in red) and DAPI (in blue). Images were acquired with a fluorescence microscope using a 40× objective.

Western blots were carried out on extracts from melanoma cell lines sensitive (A375, SKMEL28, MeWo, WM9S) or rendered resistant to chemotherapy (A375RIV, SKMELV3, MeWoR, MW9R) (Figure 1A), from melanoma cells derived from patients sensitive (Patient#1S) or resistant (Patient#1R, Patient#2R) to chemotherapy (Figure 1B), and from melanocytes (Figure 1C). In good agreement with our previous analysis performed on data from cutaneous melanoma tumors from patients [12], we observed a strong expression of Ptch1 in all the melanoma cell lines and the patients cells studied, while melanocytes do not express Ptch1. Fluorescence microscopy confirmed the expression of Ptch1 in MeWo and A375 cells (Figure 1D).

### 2.2. Methiothepin Inhibits Doxorubicin Efflux in Melanoma Cells

**Figure 2:**
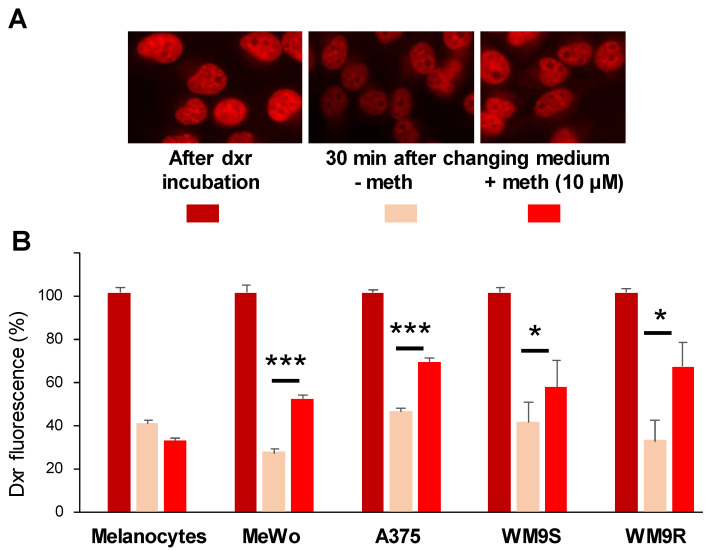
Methiothepin inhibits the doxorubicin efflux activity of Ptch1. Cells from melanocytes, melanoma cell cultures, and melanoma cell lines were seeded on coverslips and incubated with doxorubicin (dxr). After 2 h’ incubation, three coverslips were fixed for dxr loading control. The other coverslips (a triplicate per condition) were incubated with DMSO or methiothepin (meth) for 30 min and fixed. Images of each coverslip were acquired with a fluorescence microscope using a 40× objective. (**A**) Example of images obtained on MeWo cells. (**B**) Dxr fluorescence was quantified using ImageJ software for about 100 cells per condition per experiment. Histograms represent the mean ± SEM of at least three independent experiments and were analyzed using ANOVA multiple comparison test and Bonferroni correction. Significance was attained at *p* < 0.05 (*). ***: *p* < 0.0005.

We previously identified methiothepin (P375) in the screening of the Prestwick drug library on yeast-expressing human Ptch1, looking for the inhibition of the resistance to doxorubicin (dxr) conferred by Ptch1 to yeast. We took advantage of the natural fluorescence of dxr to measure the effect of methiothepin on dxr efflux in yeast by fluorescence microscopy. We showed that after incubation of yeast with dxr and removing dxr from the buffer, the dxr fluorescence measured in yeast-expressing Ptch1 was significantly higher when methiothepin was present in the efflux buffer, while this molecule had no significant effect on the dxr fluorescence of the control yeast, indicating that methiothepin specifically inhibited Ptch1 dxr efflux activity [11]. As shown in Figure 2A, incubation of MeWo cells with dxr induces an accumulation of dxr in the nuclei. The amount of dxr accumulated was drastically reduced after 30 min of incubation with a physiological buffer, and the presence of methiothepin in the buffer allowed the cells to retain a significant amount of dxr. This was also observed with A375, WM9S, and WM9R cells but not with melanocytes which do not express Ptch1 (Figure 2B). These results indicate that methiothepin inhibited the efflux of dxr from melanoma cells expressing Ptch1, and, of particular interest, from melanoma cells resistant to chemotherapy such as WM9R.

### 2.3. Methiothepin Directly Interacts with Ptch1

**Figure 3:**
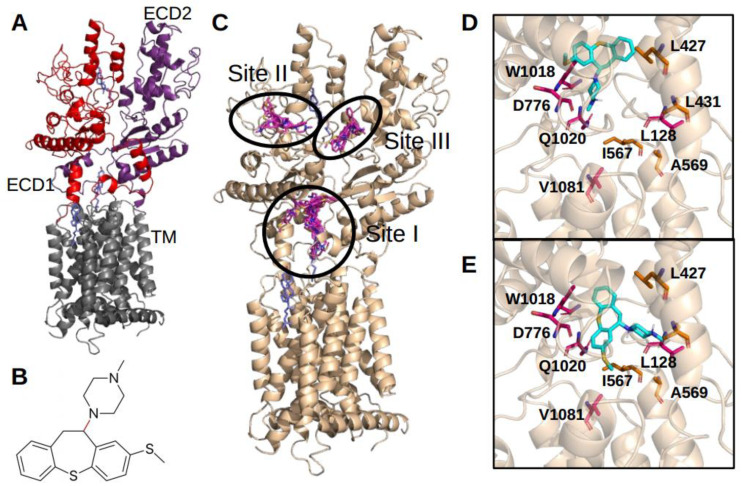
Methiothepin docking on Ptch1 structure. (**A**) Structure of Ptch1 (pdb id: 6n7h chain A). Gray: Transmembrane Domain (TM); Red: Extracellular Domain 1 (ECD1); Purple: Extracellular Domain 2 (ECD2). Cholesterol is represented in blue. (**B**) Two dimensional structure of Methiothepin. This molecule possesses only one rotatable bond represented in red. (**C**) Most represented docking clusters (in magenta). (**D**,**E**) Second best docking pose from docking targeting only site I and third best pose, respectively. Methiothepin is represented in cyan, amino-acids in magenta induce damaging phenotype when mutated, amino-acids in orange are highly conserved within Ptch proteins.

We performed a molecular docking on the structure of Ptch1 chain A (pdb 6n7h) [16]. This structure was chosen because it has the largest central cavity for cholesterol, as explained in previous studies [12,15]. This cavity is localized between the two extracellular domains (ECD) toward the transmembrane domain (TM) (Figure 3A) and cholesterol was resolved in this cavity in several of the Ptch1 structures published.

A first docking was done with a grid covering the whole structure of Ptch1 in order to identify possible binding sites. Methiothepin is a rather rigid ligand, as it possesses only one rotatable bond surrounded by cycles (Figure 3B) that may reduce its ability to accommodate in a binding pocket. However, three main binding sites for methiothepin on the Ptch1 structure were identified (Figure 3C): one which overlaps with the central cavity (site I), another in between loops of the extracellular domain 1 (site II), and the last in between both extracellular domains (site III). Sites II and III do not overlap with any identified binding sites for the cholesterol in the available structures. Note that we performed a semi-rigid docking (rigid receptor and flexible ligand), so other binding sites might exist but cannot be detected in the used structure of Ptch1.

The predicted binding energy of the best pose in each site is −8.4 kcal·mol^−1^ for site I, −7.4 kcal·mol^−1^ for site II, and −7.2 kcal·mol^−1^ for site III. As the highest predicted binding energy is for the central cavity and it correlates with results obtained with other Ptch1 drug efflux inhibitors [12,15], we decided to focus on the interactions found in this specific site only. Two of the binding poses with the lowest predicted binding energy (Figure 3D and E) are close to key amino-acids previously highlighted as interacting with other inhibitors of Ptch1 or cholesterol, and either are responsible of damaging phenotypes when mutated or highly conserved within the Ptch family proteins (L128, L427, L431, I567, D776, W1018, Q1020, V1081).

**Figure 4:**
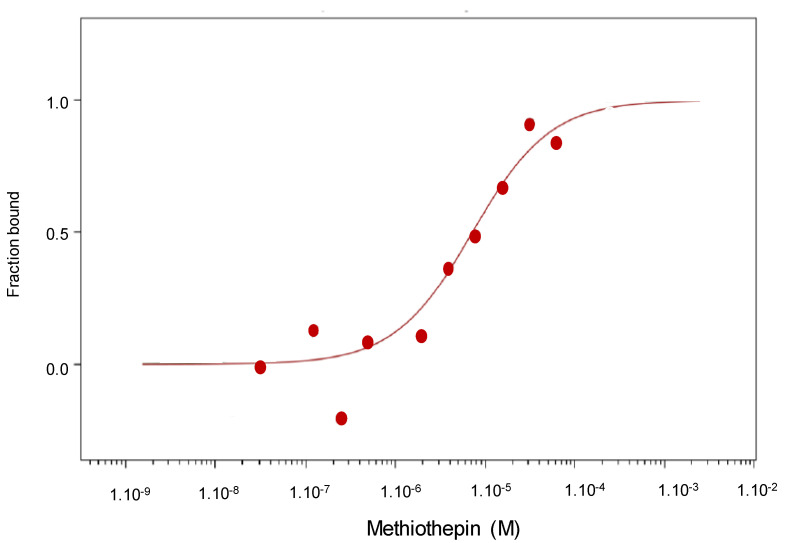
Direct binding of methiothepin to Ptch1. A total of 30 µg/mL of membranes from yeast-expressing hPtch1 protein were solubilized and incubated with 20 nM of tris-NTA-NT647 fluorescent probe, and then with 250 to 15 nM of non-labeled methiothepin. After a short incubation, the samples were loaded into capillaries in a microscale thermophoresis (MST) analysis system (Monolith NT.115, LED: 30% & MST: Medium). Concentrations on the *x*-axis are plotted in M. A Kd of 7 µM was determined.

The direct binding of methiothepin to Ptch1 was further demonstrated using MicroScale Thermophoresis (MST) and membranes prepared from yeast-expressing human Ptch1. MST revealed that methiothepin was able to specifically interact with Ptch1 solubilized from membranes prepared from yeast-expressing Ptch1 with a Kd around 7 µM (Figure 4). No binding of methiothepin was observed using control yeast (not shown).

### 2.4. Methiothepin Enhances the Sensitivity of Melanoma Cells to Doxorubicin

**Figure 5:**
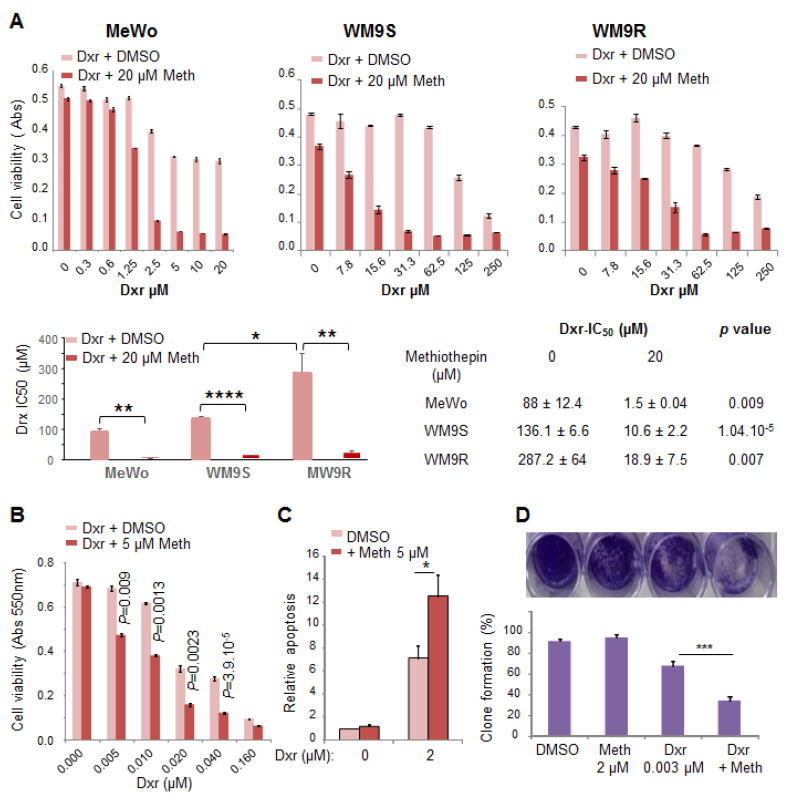
Methiothepin increases the sensitivity of melanoma cells to doxorubicin. (**A**) Methiothepin increases dxr cytotoxicity. Cell viability was measured after 24 h’ treatment with increasing concentrations of dxr with or without methiothepin (Meth) on MeWo, WM9S, and WM9R melanoma cells, and IC_50_ of dxr were calculated. Mean ± SEM of drx-IC_50_ from at least 3 experiments are reported in the histogram and table. Statistical differences between IC_50_ without and with methiothepin were calculated using the student’s t-test. Significance was attained at *p* < 0.05 (*) (**: *p* < 0.005, ****: *p* < 0.00005). (**B**) Methiothepin increases dxr anti-proliferative effect. Cell viability was measured after seven days’ treatment with increasing concentrations of dxr with or without methiothepin on MeWo melanoma cells. (**C**) Methiothepin increases dxr pro-apoptotic effect. MeWo cells apoptosis was evaluated using the luminescent assay Caspase-Glo 3/7 after 48 h’ treatment with medium alone or with medium containing dxr alone, methiothepin alone, or dxr and methiothepin together. Histograms represent the mean ± SEM of three independent experiments and data were analyzed using ANOVA multiple comparison test and Bonferroni correction. Significance was attained at *p* < 0.05 (*). (**D**) Methiothepin increased the dxr anti-clonogenic effect. Cells were seeded in 24 well-plates and treated with DMSO as control, dxr alone, methiothepin alone, or dxr and methiothepin together. After 14 days, clones were revealed with crystal violet solution, pictures were taken and absorbance was read at 550 nm after solubilization. Histograms represent the mean ± SEM of at least three independent experiments and were analyzed using ANOVA multiple comparison test and Bonferroni correction. Significance was attained at *p* < 0.05 (*) (***: *p* < 0.0005).

Confluent melanoma cells from the MeWo, WM9S, and WM9R cell lines were treated with increasing concentrations of doxorubicin (dxr), with DMSO (as control) or methiothepin for 48 h. Cell viability measured using neutral red showed that methiothepin significantly increased the cytotoxicity of dxr against these three melanoma cell lines (Figure 5A). Remarkably, our results show that WM9R cells, known to be resistant to vemurafenib, are also resistant to dxr, and that methiothepin also increases drx cytotoxicity in these resistant cells.

We also observed that the presence of methiothepin in the growth medium significantly increased the anti-proliferative effect of dxr on MeWo cells (Figure 5B). IC_50_ values of dxr calculated from these experiments are 0.021 ± 0.0025 µM in the absence of methiothepin and 0.010 ± 0.0015 µM in the presence of methiothepin with a *p* value of 0.001, indicating that this difference is significant. Moreover, quantification of caspase 3/7 activation showed that the addition of methiothepin to dxr treatment significantly increased the percentage of apoptotic MeWo cells by a factor of two (Figure 5C). Note that the concentration of methiothepin used did not affect by itself MeWo cell proliferation or apoptosis. As presented in Figure 5D, the combination of dxr and methiothepin significantly inhibited the ability of MeWo cells to form clones at a concentration of dxr or methiothepin that did not affect by itself.

Our results clearly show that methiothepin significantly increased the cytotoxic, anti-proliferative, pro-apoptotic, and anti-clonogenic effects of dxr against melanoma cells.

### 2.5. Methiothepin Enhances the Sensitivity of Melanoma Cells to Cisplatin and Oxaliplatin

**Figure 6:**
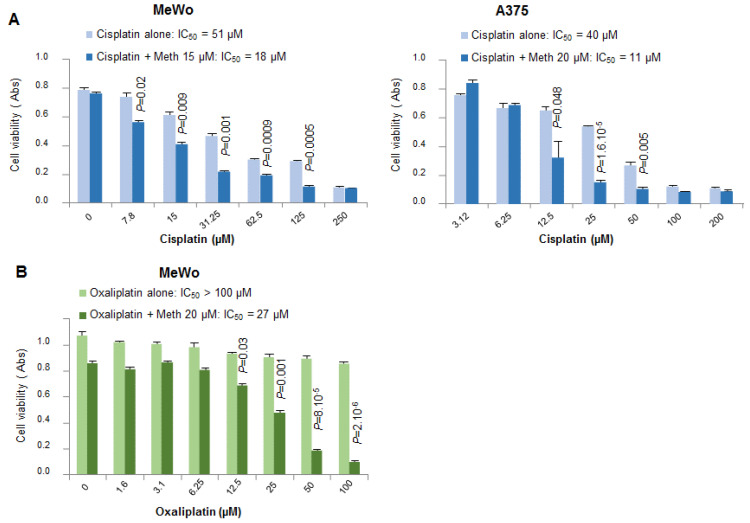
Methiothepin increases the sensitivity of melanoma cells to cisplatin and oxaliplatin. Cell viability was measured after 48 h’ treatment with increasing concentration of cisplatin (**A**) or oxaliplatin (**B**) with or without methiothepin (Meth) on MeWo and A375 melanoma cells. Statistical differences of cell viability without and with methiothepin for each concentration of cisplatin or oxaliplatin were calculated using the student’t *t-test*. The difference was statistically significant for a *p* value < 0.05. IC_50_ values of cisplatin or oxaliplatin have been calculated in the absence and the presence of methiothepin.

We tested the effect of methiothepin on the cytotoxicity of other well-known chemotherapeutic drugs such as cisplatin and oxaliplatin, which are also used for the treatment of numerous cancers. These drugs interfere with DNA repair mechanisms causing DNA damage and subsequently inducing apoptosis in cancer cells [17]. As shown in Figure 6, the addition of methiothepin strongly increased the cytotoxic effect of cisplatin and oxaliplatin against melanoma cells, leading to a significant decrease of the IC_50_ of each drug.

### 2.6. Methiothepin Enhances the Sensitivity of BRAF^V600E^ Melanoma Cells to Targeted Therapies

Vemurafenib is a targeted chemotherapy which interrupts the BRAF/MEK step in the BRAF/MEK/ERK pathway when BRAF has the V600E mutation [18,19]. A375, WM9S and WM9R melanoma cells carrying the BRAF^V600E^ mutation were treated with increasing concentrations of vemurafenib, with or without methiothepin for 24 h before assessment of cell viability.

**Figure 7:**
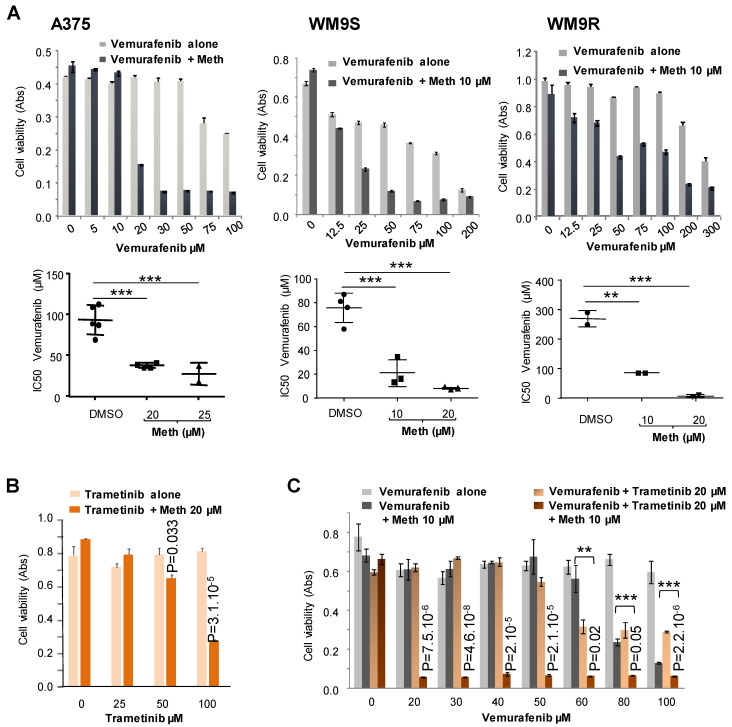
Methiothepin increases the sensitivity of BRAF^V600E^ melanoma cells to kinase inhibitors. (**A**) Methiothepin increased vemurafenib cytotoxicity on BRAF^V600E^ melanoma cells. Cell viability was measured after 24 h’ treatment with increasing concentrations of vemurafenib with or without methiothepin (meth) on A375 cells, WM9S cells, and WM9R cells rendered resistant to vemurafenib. Vemurafenib IC_50_ calculated in the absence or the presence of methiothepin are reported. Data presented are the mean ± SEM of three independent experiments. Significance was attained at *p* < 0.05 (**: *p* < 0.005, ***: *p* < 0.0005). (**B**) Methiothepin increases trametinib cytotoxicity. Cell viability measured after 48 h’ treatment with increasing concentrations of trametinib with or without methiothepin on A375 cells. Statistical differences of cell viability without and with methiothepin for each concentration of trametinib were calculated using the student’s *t-test*. The difference was considered statistically significant for a *p* value < 0.05. (**C**) Methiothepin strongly increases the cytotoxicity of the combination of kinase inhibitors on BRAF^V600E^ melanoma cells. Cell viability was measured after 48 h’ treatment with increasing concentrations of vemurafenib with or without trametinib and methiothepin (meth) on A375 cells. Statistical differences of cell viability were calculated using the student’s *t-test* for vemurafenib/trametinib combinations without and with methiothepin, and for vemurafenib/methiothepin combination in the presence and in the absence of trametinib. The difference was statistically significant for a *p* value < 0.05 (**: *p* < 0.005, ***: *p* < 0.0005).

Results show that methiothepin significantly increased the cytotoxic effect of vemurafenib against these three cell lines (Figure 7A). We observed that the effect of methiothepin was even stronger in BRAF^V600E^ melanoma cells rendered resistant to vemurafenib (WM9R) in which the IC_50_ of vemurafenib was decreased by a factor of about 25 in the presence of methiothepin. We also observed that methiothepin increased the cytotoxicity of the inhibitor of MEK kinase trametinib (Figure 7B), and of the combined treatment vemurafenib/trametinib, which is currently used on patients with BRAF^V600E^ melanoma (Figure 7C).

**Figure 8:**
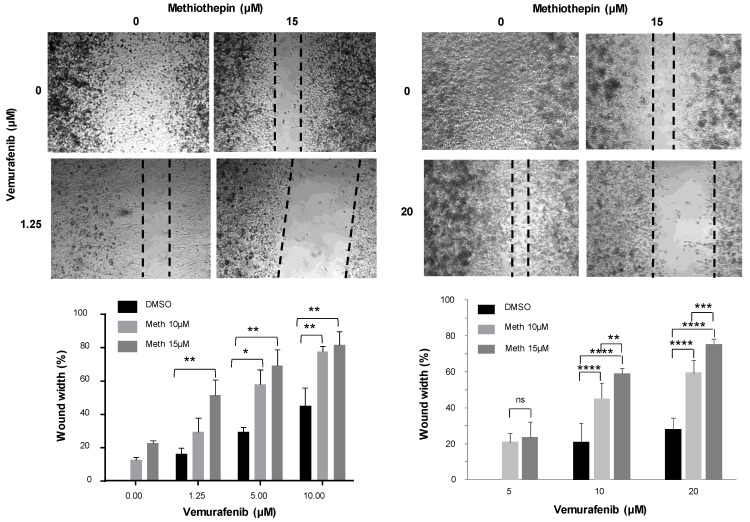
Methiothepin increases vemurafenib effect on cell migration. Migration was measured using a wound-healing assay. A wound was performed on A375 and WM9R confluent cells seeded in 24 well plates. The medium was replaced by fresh medium containing increasing concentrations of vemurafenib in the absence of methiothepin (meth), or in the presence of 10 or 15 µM of methiothepin. Two pictures were taken at two different points of each well immediately after wound, and 48 h after wound with a 5× objective. The width of the wound was measured using ImageJ software and reported as final wound width/initial wound width in percentage. Data presented are the mean ± SEM of three independent experiments. Significance was attained at *p* < 0.05 (*) (**: *p* < 0.005, ***: *p* < 0.0005, ****: *p* < 0.00005).

Wound-healing assays revealed that methiothepin also increased vemurafenib efficacy against the cells’ ability to migrate. Indeed, 48 h after performing a wound on a mat of melanoma cells, the closing area was significantly larger when methiothepin was added to vemurafenib indicating that the presence of methiothepin delays or inhibits the closure of the wound and therefore the migration of cells (Figure 8). Remarkably, this was also the case with WM9R melanoma cells resistant to treatment. These results suggest that the combination of methiothepin and vemurafenib more significantly inhibited the cell migration than vemurafenib alone even on resistant melanoma cells.

## 3. Discussion

Although significant progress has been made in therapeutic approaches, cutaneous melanoma is still a major problem worldwide, due to its high incidence and the lack of a curative treatment for advanced stages. The discovery of new molecules for treating advanced melanomas that are resistant to existing therapies is paramount to further improve patient outcomes.

We recently reported that the Hedgehog receptor Ptch1 is strongly expressed in metastatic samples from a cohort of melanoma patients, and that a high expression level of Ptch1 in patient samples significantly correlated with a lower overall survival time [12]. Accordingly, we observed that Ptch1 is endogenously expressed in various melanoma cell lines carrying or not a BRAF mutation. We previously found that decreased Ptch1 expression in MeWo and A375 melanoma cells using silencing RNA against Ptch1 strongly inhibited the efflux of doxorubicin, indicating that Ptch1 is involved in doxorubicin efflux in melanoma cells carrying or not the BRAF mutation [12]. These observations allowed us to propose Ptch1 as a new target to fight melanoma resistant to treatment, recurrence and metastases.

We then performed a screening of several chemical libraries on yeast-expressing human Ptch1, looking for the inhibition of the resistance to doxorubicin conferred by Ptch1 to yeast, and identified three molecules (panicein A hydroquinone, methiothepin, and astemizole). We showed that these molecules specifically inhibited the efflux of doxorubicin from yeast-expressing Ptch1 [11,14,15]. We recently provided evidence that panicein A hydroquinone not only increased the effect of classical chemotherapeutic treatments such as doxorubicin and cisplatin but also enhanced the effect of the BRAF inhibitor vemurafenib against BRAF mutated melanoma cells in vitro and in vivo [12]. Here, we report that methiothepin, which has been shown to increase the efficacy of doxorubicin against adrenocortical carcinoma [11], clearly exhibits the same effect as panicein A hydroquinone on melanoma cells. Indeed, our results demonstrate that the binding of methiothepin to Ptch1 increases the cytotoxicity of five different chemotherapeutic drugs (doxorubicin, cisplatin, oxaliplatin, vemurafenib and trametinib) against various melanoma cell lines carrying or not a BRAF mutation. We observed that the addition of methiothepin to vemurafenib prevents cell migration more efficiently than vemurafenib alone. Remarkably, these effects have also been observed on melanoma cells resistant to vemurafenib, indicating that the addition of methiothepin to vemurafenib allows vemurafenib to eliminate resistant melanoma cells and potentially the relapse of the primary tumor and the formation of metastases. Moreover, these effects were obtained at a concentration of chemotherapeutic drugs or methiothepin that did not affect by itself, suggesting that the use of methiothepin could allow to decrease the concentration of chemotherapeutic agents, and, therefore, of side effects for patients.

Experiments performed using microscale thermophorese technology allowed us to demonstrate that methiothepin directly interacts with Ptch1 with a Kd of 7 µM as it is the case for panicein A hydroquinone [12]. Remarkably, our docking results suggest that methiothepin binds to Ptch1 in the central cavity previously shown to bind cholesterol, like panicein A hydroquinone, and possibly on two other sites from Ptch1 structure. Interestingly, the binding of methiothepin in site III would allow to prevent the scissor movement described previously as a possible mechanism of cholesterol efflux [20].

Ptch1, as other efflux pumps from the RND family, uses the proton motive force to efflux drugs. Thus, Ptch1 transports drugs out of cells by interacting with protons from the extracellular medium [10,21]. This is only possible in tumors that have an acidic extracellular pH due to high glucose consumption of cancer cells (Warburg effect) [22]. This metabolic feature makes Ptch1 drug efflux activity specific to cancer cells. Consequently, Ptch1 drug efflux inhibitors can increase the concentration of chemotherapeutic agents only in cancer cells where the extracellular pH is more acidic than the intracellular one, and not in healthy cells where the extracellular medium is slightly more basic than the intracellular one preventing the formation of a proton entry gradient. A proof of the specificity of methiothepin to inhibit Ptch1 drug efflux activity only in cancer cells was brought by the quantification of doxorubicin in the tumors and the hearts of mice xenografted with adrenocortical carcinoma cells [11]. Actually, we observed that the amount of doxorubicin in the tumors of mice treated with the combination of methiothepin and doxorubicin was significantly higher (three times) than in tumors from mice treated with doxorubicin alone, which was not the case in the heart of animals. From these results, for melanoma treatment, we can expect that methiothepin will increase chemotherapy concentration and efficacy only in melanoma cells and not in healthy tissues preventing the increase of side effects such as those observed in clinical trials with inhibitors of ABC transporters.

## 4. Materials and Methods

### 4.1. Chemical and Biological Material

Doxorubicin hydrochloride, cisplatin, and oxaliplatin were purchased from Sigma-Aldrich, St. Louis, MO, USA. Vemurafenib and Trametinib were purchased from Selleckchem. Methiothepin maleate (P375) was purchased from Santa Cruz (CAS number: 20229-30-5; MW: 472.62).

Human melanoma cell lines A375 and MeWo were purchased from ATCC. Melanocytes, other melanoma cell lines and melanoma cell cultures from patients (Patient#1S, Patient#1R and Patient#2R) were provided Robert Ballotti (C3M, Nice, France). All cells were cultured in DMEM medium supplemented with 10% fetal bovine serum and penicillin/streptomycin (Invitrogen, Villebon sur Yvette, France) at 37 °C in a 5% CO_2_/95% air water-saturated atmosphere.

### 4.2. SDS-PAGE and Western Blotting

Total RIPA extracts were prepared from melanoma cells grown to 80% confluence in 6-well plates. Protein concentrations were determined by the DC Protein Assay (Bio-Rad, Hercules, CA, USA). Samples (50 to 80 µg) were separated on SDS-PAGE and transferred to nitrocellulose membranes (Amersham, Courtaboeuf, France) using standard techniques. After 1 h at room temperature in blocking buffer (5% non-fat milk in PBS containing 0.1% Tween-20), nitrocellulose membranes were incubated overnight at 4 °C with rabbit anti-Patched antibody (Abcam ab53715; 1/1000, Cambridge, UK) or mouse anti-β-tubulin antibody (Sigma; 1/1000). After 3 washes, membranes were incubated for 45 min with anti-rabbit (1:2000) or anti-mouse (1:5000) immunoglobulin coupled to horseradish peroxidase (Dako, Courtaboeuf, France). Detection was carried out with an ECL Prime Western Blotting detection reagent (Amersham, Courtaboeuf, France) on a Fusion FX imager (Vilber Lourmat, Collegien, France), and analyses were performed using ImageJ software.

### 4.3. Immunofluorescence

Cells were seeded on coverslips in 24-well plates and grown to 80% confluence. Coverslips were washed twice with PBS, incubated 15 min with 4% paraformaldehyde (PFA), and then 20 min with PBS/Triton 0.1% to permeabilize cells. After blocking 30 min in PBS/2% BSA, slices were incubated at 4 °C overnight with rabbit anti-Patched antibody (Abcam ab53715; 1/1000) in PBS/0.1% BSA. After three washes in PBS/0.1% BSA, slides were incubated at room temperature during 1 h with a secondary anti-rabbit antibody coupled to Alexa 594 in PBS/0.1% BSA and washed three times before mounting using antifade reagent containing DAPI (SlowFade Gold Invitrogen, Villebon sur Yvette, France) to stain nuclei. Images were acquired with a Zeiss Axioplan 2 fluorescence microscope coupled to a digital charge-coupled device camera using a 40×/1.3 Plan NeoFluar objective and filters for Alexa 594.

### 4.4. Efflux Measurements

Dxr efflux measurements were carried out as previously described [11]. Cells were seeded on coverslips in 24-well plates (Falcon, Fisher Sci. Illkirch, France) and grown to 80% confluence. Coverslips were incubated at 37 °C and 5% CO_2_ with 10 μM dxr in physiological buffer (140 mM NaCl, 5 mM KCl, 1 mM CaCl_2_, 1 mM MgSO_4_, 5 mM glucose, 20 mM HEPES, pH 7.4). After 2 h, three coverslips were immediately fixed with 4% PFA for the dxr loading control, rapidly washed with PBS and mounted in SlowFade Gold antifade reagent with DAPI (Invitrogen, Villebon sur Yvette, France). The other coverslips (triplicate per condition) were incubated with physiological buffer supplemented with DMSO or 10 µM of methiothepin under gentle shaking at room temperature and protected from light. After 30 min, coverslips were fixed with 4% PFA, washed and mounted as described above. Images were acquired with a Zeiss Axioplan 2 fluorescence microscope coupled to a digital charge-coupled device camera using a 40×/1.3 Plan NeoFluar objective and filters for Alexa 594. Dxr fluorescence was quantified using ImageJ software. Sampling of cells was performed randomly. About 100 cells (from three wells) were scored per condition per experiment.

### 4.5. Molecular Docking of Methiothepin

Semi-rigid docking of methiothepin on the Ptch1 structure was performed using the Vina toolkit [23] in USCF Chimera [24], as previously described [12,15]. The same structure of Ptch1 (pdb id 6n7h chain A) and parameters for Vina were used. Possible binding sites were first assessed performing the molecular docking with a grid including the whole structure of Ptch1. After obtaining 100 poses, clusters with at least 10% of the poses (i.e., more than 10 poses) have been identified as a binding site. Then, a docking targeting specifically the binding site of interest has been performed on a grid comprising the binding site. Finally, the best poses were analyzed by assessing the distance with the key amino acids L128, L427, L431, I567, D776, W1018, Q1020, V1081.

### 4.6. Microscale Thermophoresis

MicroScale Thermophoresis (MST) is a biophysical technique that measures the strength of the interaction between two molecules by detecting a variation in the fluorescence signal of a fluorescently labeled target as a result of an IR-laser induced temperature change. The range of the variation in the fluorescence signal correlates with the binding of a ligand to the fluorescent target. Experiments were carried out, as previously described [12]. Membranes from yeast-expressing human Ptch1 were solubilized with dodecyl maltoside (DDM) and incubated at 30 µg/mL with 20 nM of the fluorescent dye NT-647 2nd gen (NanoTemper Technologies, München, Germany) to label the His-tag present at the c-terminus of Ptch1 protein. The concentration of labeled solubilized fraction was kept constant, while the concentration of non-labeled ligand methiothepin was varied between 250 µM and 15 nM. The assay was performed in PBS containing 0.5% DMSO and 0.1% DDM. After a short incubation, the samples were loaded into Monolith™ NT.115 standard treated capillaries from NanoTemper Technologies and the MST analysis was performed using the NanoTemper Technologies Monolith NT.115 (LED: 30% and MST: Medium). The fluorescence within the capillary is excited and detected through the same objective. A focused InfraRed laser is used to locally heat a defined sample volume. The MST signal of fluorescent proteins changes upon binding to methiothepin resulting in different MST traces. Titration of methiothepin results in a gradual change in MST signal, which is plotted against the methiothepin concentration to yield a dose-response curve, which has been fitted to derive the methiothepin binding constant (Kd).

### 4.7. Cytotoxicity Assays

Cells were seeded in 96-well plates (Falcon Fisher Scientific Illkirch, France) in triplicate and grown in medium to achieve 70% to 80% confluence. Medium was then removed and replaced with 100 µL/well of complete medium containing methiothepin or DMSO as a control. After 2 h, 100 µL of complete medium containing serial dilutions of dxr, cisplatin, oxaliplatin, vemurafenib or trametinib were added. Plates were incubated at 37 °C and 5% CO_2_. After 24 or 48 h, cells were incubated for 3 h at 37 °C with 100 µL/well neutral red (NR) solution (50 µg/mL in medium) following the manufacturer’s protocol. Absorbance was measured in a microplate reader (Multiskan Go Microplate Spectrophotometer from Thermo Scientific, Waltham, MA, USA). IC_50_, defined as the concentration that resulted in a 50% decrease in the number of live cells, were calculated using GraphPad Prism 6 software.

### 4.8. Apoptosis Measurements

Cells were seeded at a density of 7000 cells per well in a 96-well white polystyrene plate (Falcon) in triplicate and cultured overnight at 37 °C and 5% CO_2_. After removal of the medium, cells were treated 48 h with medium alone or with medium containing DMSO (control), dxr alone, methiothepin alone or dxr and methiothepin together. Quantification of caspase 3/7 activity was performed using the luminescent assay CaspaseGlo 3/7 (Promega, Charbonnières-les-Bains, France) and a luminometer (Glomax 96 Microplate Luminometer (Promega, France) following the manufacturer’s protocol.

### 4.9. Proliferation

Cells were seeded at a density of 5000 cells per well in 96-well plates (Falcon) in triplicate and grown for 24 h at 37 °C and 5% CO_2_. After removal of the medium, cells were treated with medium containing serial dilutions of dxr in the presence of DMSO (control) or methiothepin.

After 7 days at 37 °C and 5% CO_2_, NR assay was performed for quantification of living cells. IC_50_ values were calculated using GraphPad Prism 6 software.

### 4.10. Clone Formation

Cells were seeded in 24-well plates (Falcon) at a density of 5000 cells per well in triplicate, treated with DMSO as control, methiothepin alone, dxr alone or a combination of methiothepin and dxr, and incubated at 37 °C and 5% CO_2_. After 7 days, 500 µL of medium containing the respective molecules was added to the wells to maintain the medium volume in wells. After 14 days, cells were fixed with 4% PFA and incubated with 0.4% crystal violet. After 1 h, cells were washed quickly with PBS and pictures of each well were taken. Cells were then solubilized with 1% SDS and absorbance was read in a microplate reader at 550 nm (Multiskan Go Microplate Spectrophotometer, Thermo Scientific).

### 4.11. Wound-Healing Assay

Once cells were confluent in 24-well plates, a wound was performed with a p200 tip. Wells were washed with PBS to remove cells in suspension and filled with medium containing DMSO (control), vemurafenib alone, methiothepin alone or vemurafenib and methiothepin. Two pictures were taken at two different points of each well immediately after wounding, and 48 h after wounding with Leica DM IRB (5×). The width of the wound was measured using ImageJ software and reported as percentage final wound width/initial wound width.

### 4.12. Statistical Analysis

All results represent at least three independent replications. Data are shown as the mean value ± SEM. Prism 6 (GraphPad) was used to determine IC_50_ values and other statistical analyses using one-way analysis of variance (ANOVA) followed by Bonferroni’s Multiple Comparison Tests.

## 5. Conclusions

The present study provides one more proof of concept that the use of inhibitors of Ptch1 drug efflux activity as adjuvant to classical or targeted chemotherapy could be a novel way to circumvent drug resistance, recurrence and metastasis of tumors expressing Ptch1 with very limited side effects for patients.

## Data Availability

The data presented in this study are available on request from the corresponding author.

## Sample Availability

Samples of the compounds methiothepin are not available from the authors.
